# Determination of Catechin and Epicatechin Content in Chocolates by High-Performance Liquid Chromatography

**DOI:** 10.1155/2014/628196

**Published:** 2014-10-28

**Authors:** Raju V. S. S. Gottumukkala, Nareshraju Nadimpalli, Kannababu Sukala, Gottumukkala V. Subbaraju

**Affiliations:** Natsol Laboratories Private Limited, 2nd Floor, Research & Development Building, Ramky Commercial Hub, Jawaharlal Nehru Pharma City, Visakhapatnam 531 019, India

## Abstract

A simple and sensitive reversed phase high-performance liquid chromatographic (HPLC) method has been developed for the determination of catechin and epicatechin in cocoa powder and chocolates. The separation was achieved on a reversed phase C 18 column (TARGA) 5 *μ*m by gradient elution with a flow rate of 1.0 mL/minute with an operating temperature of 30°C and detection with a UV-Visible detector was at 280 nm. The method was validated for linearity, precision, intra- and interday precision, and accuracy. The developed method is successfully applied for the determination of catechin and epicatechin content in chocolates. The Godiva brand chocolate contains high concentration of epicatechin.

## 1. Introduction

Dietary polyphenols comprise a wide range of aromatic compounds. The polyphenols that are present in foods can be divided broadly into two main groups: flavonoids and other compounds that are mostly monocyclic aromatic acids. Flavonoids contain two phenolic moieties and a pyran unit [1]. Catechin (1) and epicatechin (2) belong to flavonoids.

Cocoa and chocolate products have the highest concentration of flavonoids among commonly consumed foods and also have many health promoting properties. Over 10 percent of the weight of cocoa powder is flavonoids. Cocoa and chocolate are among the most concentrated sources of the procyanidin flavonoids, catechin, and epicatechin. For example, dark chocolate has been found to be very nutritious, potent antioxidant, that improves blood flow, lowers blood pressure, raises HDL, protects LDL against oxidation, lowers the risk of cardiovascular disease, protects the skin against the sun, and improves blood flow to the brain.

The active ingredient of dark chocolate has been identified as epicatechin [2]. Epicatechin has proved that consumption of plain, dark chocolate resulted in an increase in both the total antioxidant capacity and the (–) epicatechin content of blood plasma but that these effects are markedly reduced when the chocolate is consumed with milk or if milk is incorporated as milk chocolate. A study conducted over a two-week period in 21 healthy adult subjects with a daily consumption of high-flavonoid (213 mg procyanidins, 46 mg epicatechin) or low-flavonoid dark chocolate bars (46 g, 1.6 oz) observed improvement in endothelial function associated with an increase in plasma epicatechin concentrations and no changes in oxidative stress measures, lipid profiles, blood pressure, body weight or BMI were observed. Epicatechin has also been proved to improve vascular functions and insulin sensitivity, to reduce blood pressure and platelet reactivity, and to be good antioxidant [3–7].

The growing interest in these compounds and the number of methods for quantification in various herbs, herbal extracts, herbal formulations, and food items have been developed. A thorough literature search revealed that the high-performance liquid chromatographic (HPLC) methods are more predominant. Several HPLC methods with different detection modes, ultraviolet [8–13], photodiode array [14, 15], electrochemical [10, 16, 17], capillary electrophoresis [18, 19], fluorescence [10, 20], and mass [21, 22] detection, as well as few high-performance thin layer chromatographic (HPTLC) methods [23, 24], are also reported.

In view of the growing interest in epicatechin, we report a simple, rapid, and precise gradient HPLC method with an economical mobile phase for the simultaneous separation and quantification of catechin and epicatechin, in cocoa powder and chocolates. The present paper describes the method of determination of catechin and epicatechin.

## 2. Materials and Methods

### 2.1. Commercial Samples

Commercial chocolates were procured from USA and from local market.

### 2.2. Reagents and Chemicals

All HPLC grade solvents (acetonitrile, methanol, water, and orthophosphoric acid) were purchased from Qualigens (Mumbai, India). Standard catechin and epicatechins were prepared by Natsol Laboratories Private Limited, Visakhapatnam, India. Identity and purity (>98%) of the compounds were confirmed by chromatographic (HPLC) and spectral (IR, Mass, and NMR) data.

### 2.3. HPLC Instrumentation and Conditions

The HPLC system (Shimadzu, 2010 CHT) consisted of quaternary pump with vacuum degasser, thermostatted column compartment, autosampler, and UV detector. A reverse-phase column (TARGA, C18, 5 *μ*, 250 × 4.6 mm) was used and the column temperature was maintained at 30°C. HPLC mobile phase was prepared as follows. Solution A: 0.1 mL of orthophosphoric acid dissolved in 900 mL of HPLC grade water and the volume was made up to 1000 mL with water and the solution was filtered through 0.45 *μ*m membrane filter and degassed in a sonicator for 3 minutes, Solution B: acetonitrile. Mobile phase was run using gradient elution: at the time 0.01 minutes 11% B; at the time 30 minutes 25% B; at the time 35 to 39 minutes 100% B; and at the time 40 to 50 minutes 11% B. The mobile phase flow rate was 1.0 mL/minute and the injection volume was 10 *μ*L. The eluents were detected and analyzed at 280 nm.

### 2.4. Preparation of Standard Solutions

Stock solutions of catechin and epicatechin (4 mg/mL) were prepared by dissolving standards into methanol. Less concentrated solutions were prepared, as needed by dilution with methanol.

### 2.5. Preparation of Samples

#### 2.5.1. Cocoa Powder

50 mg of the sample was transferred into a 100 mL volumetric flask containing 75 mL of methanol, sonicated for 10 minutes, diluted to 100 mL with methanol, and filtered through 0.45 *μ*m membrane filter.

#### 2.5.2. Chocolates

About 10 gm of chocolate sample was extracted with methanol on a hot water bath till the colorless methanolic extract was obtained. Combine all the methanolic fractions and filter using Whatman no. 1 filter paper. Evaporate the combined filtered extract to 100 mL on a roto evaporator. Filter this solution through 0.45 *μ*m membrane filter.

### 2.6. Calibration

Calibration standards were prepared by diluting the catechin and epicatechin stock solutions with methanol in the concentration range of 100–600 *μ*g/mL. The standard curves were obtained by using the peak areas of six different concentrations in six replicate assays and were expressed by the linear least square regression equation.

### 2.7. Method Validation

The specificity of the method was ascertained by analyzing the standards and the samples. The peaks for catechin and epicatechin in the samples were confirmed by comparing the retention times of the peak with those of standards.

The linearity of the method was checked with catechin and epicatechin standards with the calibration curves in the range 100–600 *μ*g/mL, using six different concentrations in six replicate assays. Limit of detection (LOD) and limit of quantification (LOQ) were obtained from the standard deviation (*σ*) of the blank response (*n* = 6) and slope (*S*) of the calibration curve using the formulae 3.3 *σ*/*S* and 10 *σ*/*S*, respectively.

Accuracy was determined by spiking preanalyzed sample with low, medium, and high concentrations in the calibration range of the standard catechin and epicatechin and analyzed by the proposed method. The experiments were conducted six times to check the recovery of the analyte at different levels in the samples.

Precision is a measure of repeatability of the analytical method in the normal operating conditions. Precision (intra- and interday) of the method was verified by six determinations of three concentrations of the standard catechin and epicatechin on the same day (intraday) and on different days (interday) which were carried out and expressed as percent relative standard deviation (%RSD).

## 3. Results and Discussion

### 3.1. Optimization of Chromatographic Conditions

A simple method was developed for the determination of catechin and epicatechin based on a reversed phase HPLC separation combined with ultraviolet detection. The gradient system was chosen to minimize the variations of the baseline, considering the simplicity, precision, and accuracy. The stability of the samples and standards of catechin and epicatechin were checked and found to be stable for 12 hours.

A systematic study conducted on the effect of column selection and mobile phase composition on the separation of catechin and epicatechin based on the resolution and peak shapes. Best results were obtained with TARGA C18 column, with 5 *μ*m particle size, 250 mm length, and 4.6 mm i.d, and a gradient system of mobile phase comprises 0.1% (v/v).

**Figure 1:**
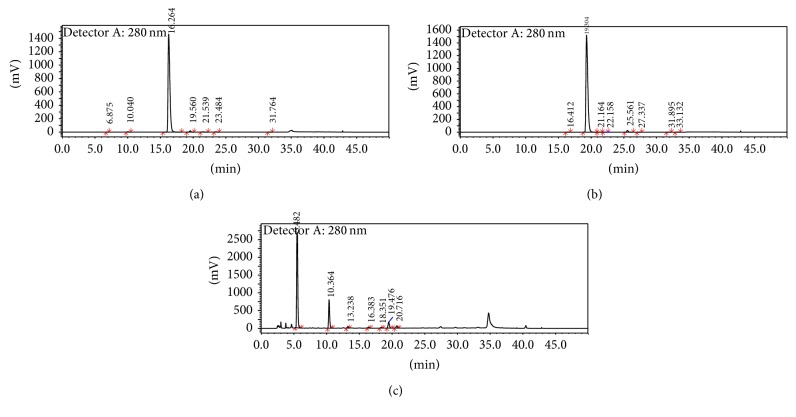
HPLC chromatograms. (a) Standard catechin (400 *μ*g/mL). (b) Standard epicatechin (400 *μ*g/mL). (c) Godiva Chocolatier 85% cacao.

Phosphoric acid in water (Mobile phase A) and acetonitrile (Mobile phase B) with 1.0 mL/minute flow rate enable the baseline separation of catechin and epicatechin within 50 minutes. All the separations were performed at 30°C, absorption measurement at 280 nm was selected, and the compounds catechin and epicatechin were effectively detected (Figure 1).

### 3.2. Validation of the Assay Method

Method for quantitative analysis of catechin and epicatechin was validated with regard to its specificity, linearity, accuracy, and precision by utilizing the ICH guidelines [25]. The specificity of the method was confirmed by reliability of compound peaks corresponding to catechin and epicatechin in the sample. The sample was spiked with standard catechin and epicatechin, individually, which were separated by gradient mobile phase system. The peak areas of the catechin and epicatechin in the sample were changed consistently with their corresponding standards. The study confirmed the stable retention times of catechin and epicatechin in the samples which were comparable to retention times of standards with ±0.2 to ±0.3 minutes of variation (Table 1).

The linearity of the method was checked with standards corresponding to catechin and epicatechin with the calibration curves in the concentration range 100–600 *μ*g/mL. The regression equation and the correlation coefficient were observed with six replicate analyses for each concentration. The results of linearity data were reported in (Table 2) showing that the correlation coefficient of the equation was >0.99 and the two calibration curves yielded straight lines in a wide range.

The limit of detection (LOD) of catechin and epicatechin was 0.012 *μ*g/mL and 0.15 *μ*g/mL, respectively, and the limit of quantification (LOQ) of the catechin and epicatechin was 0.036 *μ*g/mL and 0.45 *μ*g/mL, respectively.

Accuracy of the analytical method was determined by spiking sample with known amounts of catechin and epicatechin at three concentration levels. The samples were analyzed under optimized conditions and the recovery rates were observed in the range 99.98% to 100.24% for catechin and 99.48% to 100.26% for epicatechin (Table 3). The average recovery percentage values were found to be 100.06% and 99.91% for catechin and epicatechin.

The precision of the method was validated both for intra- and interday variation. The results depicted in (Table 4) showed that no significant intra- and interday variations were observed in the analysis of catechin and epicatechin.

### 3.3. Application of the Method

The method developed was applied to analyze cocoa powder and ten different chocolates which were procured from domestic market and United States of America. The results presented in (Table 5) reveal that epicatechin is present in higher concentrations than catechin in the cocoa powder and chocolates. The presence of excipients like sugar, milk fat, and vanilla does not interfere in the determination of catechin and epicatechin in chocolates.

## 4. Conclusions 

The growing importance of epicatechin as a constituent of therapeutic significance necessitated the development of highly sensitive and selective methods. A simple reversed phase HPLC method has been developed and the results demonstrate that the method developed was highly specific, accurate, and precise and it could be used in the determination of catechin and epicatechin content in chocolates. Interestingly, the branded chocolates contain higher levels of catechin and epicatechin. Chocolates of Indian origin contain lesser content of catechin and epicatechin comparable to the brands available in USA.

## Figures and Tables

**Table 1:** Specificity.

Compound	Retention time (minutes) standard	Retention time (minutes) Godiva Chocolatier 85% cacao
Catechin	16.264 ± 0.2	16.383 ± 0.2
Epicatechin	19.304 ± 0.2	19.476 ± 0.2

**Table 2:** Calibration, linearity, and LOD and LOQ data.

Compound	Linearity range (*μ*g/mL)	Regression equation	Correlation coefficient	LOD (*μ*g/mL)	LOQ (*μ*g/mL)
Catechin	100–600	*y* = 74777*x* + 13899	0.9990	0.012	0.15
Epicatechin	100–600	*y* = 77598*x* − 17409	0.9980	0.036	0.45

*y* = peak area; *x* = concentration of analyte.

**Table 3:** Recovery studies (*n* = 6).

Compound	Amount of compound added (*μ*g)	Amount of compound recovered (*μ*g ± S.D.)	% recovery
Catechin	200	200.48 ± 1.25	100.24
	400	399.52 ± 0.96	99.88
	600	600.51 ± 1.09	100.08

Epicatechin	200	198.95 ± 1.30	99.48
	400	401.05 ± 1.05	100.26
	600	599.85 ± 1.35	99.98

**Table 4:** Intra- and interday precision (*n* = 6).

Compound	Intraday			Interday		
	Concentration (*μ*g/mL)	Average peak area	% RSD	Concentration (*μ*g/mL)	Average peak area	% RSD
Catechin	200	1507937	0.13	200	1537115	0.20
	400	2924044	0.27	400	2968715	0.23
	600	4458083	0.33	600	4444284	0.67

Epicatechin	200	1633896	0.03	200	1609962	0.08
	400	3065181	0.04	400	3079683	0.21
	600	4699541	0.21	600	4617629	0.06

**Table 5:** Determination of catechin and epicatechin in samples (*n* = 6; % relative standard deviations are given in parenthesis).

Sample		Catechin	Epicatechin
Cocoa powder^1^		0.20% (0.72)	0.28% (0.55)

Chocolates	Serving size (gm)	Catechin per serving size	Epicatechin per serving size

Lindt Excellence, 90% cocoa^2^	40	6.04 mg (0.27)	17.32 mg (0.17)
Lindt Excellence, 85% cocoa^2^	40	4.80 mg (0.38)	22.76 mg (0.57)
Lindt Excellence, 70% cocoa^2^	40	7.84 mg (0.32)	28.56 mg (0.24)
Ghirardelli Chocolate, 86% cacao^3^	45	4.50 mg (0.51)	23.85 mg (0.28)
Ghirardelli Chocolate, 72% cacao^3^	38	5.78 mg (0.34)	27.70 mg (0.33)
Ghirardelli Chocolate, 60% cacao^3^	38	4.79 mg (0.32)	17.78 mg (0.23)
Godiva Chocolatier, 85% cacao^4^	40	5.20 mg (0.79)	31.68 mg (0.08)
Safeway Select chocolate, 85% cacao^5^	40	4.76 mg (0.83)	10.52 mg (1.04)
Safeway Select chocolate, 72% cacao^5^	40	4.84 mg (0.86)	14.64 mg (0.69)
Green & Black's chocolate, 50% cocoa^6^	40	3.28 mg (0.36)	11.92 mg (0.14)
Bournville Cranberry chocolate, 50% cocoa^7^	33	1.49 mg (1.25)	3.76 mg (0.18)
Bournville Rich Cocoa chocolate, 50% cocoa^7^	33	1.09 mg (0.27)	3.60 mg (1.81)

^1^Samples were supplied by M/S Natsol Laboratories Private Limited, Visakhapatnam, India.

^2^Lindt and Sprungli Inc., Stratham, NH 03885, USA.

^3^Ghirardelli Chocolate Company, San Leandro, CA 94578, USA.

^4^Godiva Chocolatier company, USA, product of Germany.

^5^Safeway Select Inc., P.O. Box  99, Pleasanton, CA 94566, USA, product of Switzerland.

^6^Green & Black's, Parsippany, NJ 07054, USA, made in Italy.

^7^Cadbury India Limited, Mumbai, India.
